# The Virtuous *Galleria mellonella* Model for Scientific Experimentation

**DOI:** 10.3390/antibiotics12030505

**Published:** 2023-03-03

**Authors:** Isa Serrano, Cláudia Verdial, Luís Tavares, Manuela Oliveira

**Affiliations:** 1 CIISA—Center for Interdisciplinary Research in Animal Health, Faculty of Veterinary Medicine, University of Lisbon, Avenida da Universidade Técnica, 1300-477 Lisboa, Portugal; 2 Associate Laboratory for Animal and Veterinary Sciences (AL4AnimalS), 1300-477 Lisboa, Portugal

**Keywords:** antimicrobial therapies, bacterial pathogens, drug screening, fungal pathogens, *Galleria mellonella*, infection model, innate immunity, standardization, toxicity model, 3Rs

## Abstract

The first research on the insect *Galleria mellonella* was published 85 years ago, and the larva is now widely used as a model to study infections caused by bacterial and fungal pathogens, for screening new antimicrobials, to study the adjacent immune response in co-infections or in host-pathogen interaction, as well as in a toxicity model. The immune system of the *G. mellonella* model shows remarkable similarities with mammals. Furthermore, results from *G. mellonella* correlate positively with mammalian models and with other invertebrate models. Unlike other invertebrate models, *G. mellonella* can withstand temperatures of 37 °C, and its handling and experimental procedures are simpler. Despite having some disadvantages, *G. mellonella* is a virtuous in vivo model to be used in preclinical studies, as an intermediate model between in vitro and mammalian in vivo studies, and is a great example on how to apply the bioethics principle of the 3Rs (Replacement, Reduction, and Refinement) in animal experimentation. This review aims to discuss the progress of the *G. mellonella* model, highlighting the key aspects of its use, including experimental design considerations and the necessity to standardize them. A different score in the “cocoon” category included in the *G. mellonella* Health Index Scoring System is also proposed.

## 1. Introduction

The insect larva of *Galleria mellonella* has been widely used in the last few decades as an infection model to study bacterial and fungal infections and for assessing the efficacy of novel antimicrobial drugs, before proceeding to preclinical studies in mammals, reinforcing the application of the 3Rs principles (replacement, reduction, and refinement) in animal experimentation. The *G. mellonella* model offers several advantages over mammalian models: it does not require ethical approval, it is easily manipulated, it is inexpensive, it allows large-scale breeding, and it allows a rapid experimental execution, associated with the ability to incubate caterpillars at temperatures between 25 °C and 37 °C and the simple inoculation of the pathogens to be tested [1,2,3].

Insects diverged from vertebrates approximately 500 million years ago. Although vertebrates have developed an adaptive immune response, their innate immune response still retains remarkable similarities with the immune response in insects, allowing the establishment of a positive correlation between most results obtained in *G. mellonella* and mammalian models [3,4]. A positive correlation between *G. mellonella* and other invertebrate models was also already shown, including with a *Caenorhabditis elegans* model of methicillin-resistant *Staphylococcus aureus* (MRSA) infection [5]. Furthermore, the *G. mellonella* model has been cited as suitable for the pre-screening of efficacy assessment of new antibacterial [3,6] and antifungal agents [7,8], of therapies combining new compounds with conventional antibiotics, and also a suitable model to study virulence factor expression, co-infections, host-pathogen interactions, innate immune response to microorganisms, and toxicity, with the main application in human health studies [9,10].

Historically, *G. mellonella* was described in the 4th century BC by Aristoteles as a honeybee pest in the book *Historia Animallium* (Book VIII, Chapter 27) [9]. The first publication in PubMed was in 1938, describing the genetic characteristics of *G. mellonella* following X-ray treatment [11]. Then, in 1951, this insect was used for the first time as a toxicity model for an antituberculosis drug [12]. Further studies in 1957 and 1961 used it to test antifungal agents [13,14], and since the 60s *G. mellonella* was validated as an infection model for many bacterial species, *Salmonella enterica* serovar Typhimurium being the first one [15], in addition to several fungi [16]. Although viruses can only infect specific hosts, leading researchers to use mammalian models for viruses related studies, some insect and mammalian viruses, such as the *Tipula iridescent* virus and the Nodamura virus [17,18], have been validated in the *G. mellonella* model. Since 2000, the use of the *G. mellonella* model has been exponentially increasing, being widely used for infection studies in the scope of human and veterinary pathogens [3,9]. The *G. mellonella* model was also successfully used to study the effects of co-infection in 2004 [19]. The *G. mellonella* transcriptome was sequenced in 2011 and its genome was sequenced in 2018, revealing over 14,000 genes [20]. Genome sequencing and studies on the immune response at the proteomic, epigenetic, and transcriptomic levels have launched new areas of research [21,22,23]. Its wide application is due to *G. mellonella* susceptibility to a high number of bacteria, and, according to Champion et al. [24] to twenty-nine species of fungi, seven viruses, one species of parasite, and sixteen biological toxins [24].

The first researcher who invested time in studying *G. mellonella* could not imagine the importance that it has in the present, as according to a Pubmed search over 2969 scientific articles have been published on *G. mellonella* from 1938 until today. Currently, *G. mellonella* larvae are commercially available, and practical recommendations and videos on protocols for rearing and testing are accessible.

The main purpose of this review is to discuss the development of the *G. mellonella* model, highlighting the main aspects of its use, including the necessity to standardize experimental procedures. We also propose a different score on a category included in the *G. mellonella* Health Index Scoring System [25].

## 2. Characterization of *Galleria mellonella*

The great wax moth or the honeycomb moth, *Galleria mellonella* (Linnaeus, 1758), is an insect moth from the Phylum Arthropoda, Class Insects, order Lepidoptera and family Pyralidae (snout moths) [26]. It is a ubiquitous parasite of the honeybees *Apis mellifera* and *Apis cerana* and of their hives, and it can be found in beehives, bumblebee nests, wasp nests, or in stored waxes. In nature, caterpillars feed on honey, and also pollen, beeswax, pupa skins, cocoons, and feces from the hive [27].

The *G. mellonella* larvae can cause galleriasis within the hives, a phenomenon in which the hatching larvae create tunnels through the honeycombs containing larva and honey stores. The larvae cover these tunnels with silk entangling and starving the emerging bees, leading to colony loss and a reduction in the size of the migratory bee swarms [28]. The tunnels make holes through which honey leaks out [27,28], resulting in massive destruction of the combs. *G. mellonella* adults and larvae are carriers of two viruses, the Israeli acute paralysis virus and the black queen cell virus, that can potentially infect and kill honeybees [27,28]. In Texas and Florida, where tropical climate conditions are found, approximately USD 1.5 and 3 losses per colony were recorded in 1997, respectively [29]. Due to the destructive nature of the pest, several management techniques are under study, such as mass trapping, by attracting the pest with a food component, or with a pheromone to a trap. However, maintaining good sanitation of beehives is still the most effective method in small-scale beekeeping [27].

*Galleria mellonella* is distributed worldwide, being present on all continents, especially in mountain range areas, except Antarctica [27], coinciding with the occurrence of its host bees. It has been detected in seventy-seven countries and in several islands, and it is anticipated that the pest may spread further, especially due to climate change [27].

*Galleria mellonella* is a typical holometabolous insect undergoing four developmental stages in its life cycle: the egg, larva, pupa, and adult [27,30,31]. The duration of a complete cycle is approximately six to eight weeks at 29–39 °C and high humidity [31], with four to six generations per year. Complete metamorphosis is affected by abiotic factors such as temperature, diet, and relative humidity, and by biotic factors such as competition for food and cannibalism [27,32]. The 50–150 eggs layered by the female between the cracks of the honeycomb are spheroidal-shaped and white to light pink in color. The development into larvae is temperature dependent, and lasts between three to eight days at 24–27 °C, and thirty days at 10–16 °C [33]. Moreover, worm growth could be limited when it is cultured at 4 °C. The creamy-white larvae show a reddish-brown dome head and become light gray as they grow. They measure 1–23 mm and resemble a caterpillar, presenting a tube-like structure for processing and storing food. They can remain in the larval stage from 28 days, at optimal conditions, to six months, and they undergo about 8–10 molting stages during this period [10,34].

The larva body is divided into a head, a three-segmented thorax with six legs, and an abdomen of ten segments with eight prolegs and two anal prolegs [34]. The larvae dorsal region is called the “new immune tissue” because it is where the immune response is organized [35,36]. Within the inner cavity, there is the hemolymph, which is the larval circulatory system, and the fat body, a biosynthetic organ analogous to the mammalian liver. Within the fat body, the digestive system and the silk gland can be found. The ventral region corresponds to the nervous system and contains several ganglia [34,37] 

Larvae at the last instar produce silk using their salivary glands to form cocoons that should be protected from the open air and excess humidity. At this moment, at the stage of pre-pupae, the larvae stop eating and become less mobile. Pupae are immobilized in cocoons and do not eat during this period for one to nine weeks until emerging as adults. The color of the cocoon depends upon the presence of colored pigments in the layer, varying from white to brown, and finally turning into a light golden-brown cocoon [30,31,34]. 

Adult moths’ color varies from reddish-brown to light brown. They are sensitive to light; hence, they fly mostly at nighttime and stay in the dark during the daytime, and as in the pupae stage they do not feed [27]. Many differences can be observed between male and female adult moths: males are slightly smaller, at 14–38 mm in length in wingspan, are lighter than females, and they live twenty-one days, whereas females live for only twelve days [38]. 

*Galleria mellonella* larvae are capable of digesting polyethylene, a non-biodegradable polymer, relatively quickly [39]. Bombelli et al. [39] stated that this capability may be explained by the biodegradation of the beeswax constituents, such as lipid compounds, including fatty acids, alkenes, and esters, which involves the breaking of the same kind of chemical bonds as those of polyethylene [39].

The *G. mellonella* larvae microbiota (the larvae microbial community) is dominated by a single *Enterococcus* taxon, presenting *E. gallinarum* or *E. saccharolyticus* as a main species, independently of the body site sampled, although other taxa, such as the *Staphylococcus*, *Pseudomonas* and *Enterobacter* species, were also already found [40]. Interestingly, commercially available larvae show a lower bacterial load than standardized research larvae, probably due to previous treatments with hormones and antibiotics [10,40]. However, there has been some disagreement regarding the microbial composition of *G. mellonella*, as some authors argue that it is not as simple as has been advocated [41]. According to Gohl et al. [41] the *G. mellonella* microbiome is diverse, dominated by the Proteobacteria and Firmicutes phylum, appears to have a resident microbiome represented by *Ralstonia* bacteria, and is affected by both diet and ontogeny [41].

According to Johnston and Rolff [42], the larval digestive microbiota composition changes during larval metamorphosis. In more developed stages, *Enterococcus* species are more predominant, whereas Enterobacterales and staphylococci became undetectable [42]. This constitutes valuable data, as the *G. mellonella* larvae infection model is usually experimentally used at the final instar stage. It has also been observed that diet has an impact on the microbiome [41,42,43], even though the microbiota is not needed for metabolic support in axenic *G. mellonella* (harboring no cultivatable organisms) [42,43].

## 3. Immune System of *Galleria mellonella*

As observed in mammalians, the innate immune system of insects such as *G. mellonella* comprises the cellular and the humoral immune system, and is more advanced than other invertebrates such as nematodes [44]. 

The innate immune system is non-specific and is distributed throughout the body. It is the first line of defense against microbes, maintaining homeostasis and preventing infections [45]. The lack of adaptive immunity in *G. mellonella* and other insects is in fact an advantage because it allows the study of host-pathogen interactions without the interference of adaptive responses [46]. Obviously, if the study aims to understand the response of the adaptive immune system to a certain pathogen, insect models are not adequate. The innate immune response of *G. mellonella* shares several properties with the mammalian innate immune system (for example, phagocytosis in insects and mammals is believed to be very similar [45,47], therefore *G. mellonella* is a valuable in vivo model to be used in preclinical studies as an intermediate model between in vitro and mammalian in vivo studies [2,3,9].

In *G. mellonella,* there is considerable overlap between humoral and cellular defenses, as many humoral molecules affect cellular immune response, and cellular immune response is an important source of many humoral molecules [48]. The cellular immune response is mediated by hemocytes, which are phagocytic cells that present an analogous function to mammalian blood. They are found free in the hemolymph or attached to internal organs, such as the digestive tract, fat body, and surface of the insect heart [35,49,50]. Hemocytes recognize pathogenic microorganisms through the direct interaction of their surface receptors with pathogen molecules, or indirectly by the recognition of humoral immune receptors that bind to and opsonize the pathogen. Therefore, both humoral and cell surface receptors are involved in these recognition events. These receptors are known as pathogen recognition receptors (PRRs), and are able to recognize pathogen-associated molecular patterns (PAMPs). PAMPs are conserved microbial components, including lipopolysaccharide (LPS), peptidoglycan, lipoteichoic acids (LTA), and β-1,3 glucan [9,51]. 

Hemocytes are involved in phagocytosis, encapsulation, and nodulation of the invading agent. Six types of hemocytes can be found in *G. mellonella*: plasmatocytes and granular cells, which are the most abundant hemocytes [52]; prohemocytes, which are progenitor cells that can differentiate into several cell types; coagulocytes, involved in hemolymph coagulation; spherulocytes, which transport and secrete several cuticular components; and oenocytoids, which are involved in the melanization pathway and, like mammal neutrophils, are able to secrete extracellular nucleic acid traps involved in pathogen sequestration and coagulation activation [2,10]. Encapsulation takes place when pathogens are too large to be phagocytosed and occurs without melanization. Nodulation begins with the attachment of granular cells to the microbes’ surface, triggering the release of multiple plasmatocyte spreading peptides, which will attach around the bacteria, fungal spores’ surface, or foreign targets, resulting in the formation of a smooth capsule [53,54]. This step is often followed by melanization [3].

The hemocytes’ concentration varies in response to pathogenic agents and during the *G. mellonella* life cycle [35,49,50]. Just after infection, there is an activation of the larval immune system regardless of the pathogenic agent, which may lead to a decrease in hemocyte count [55,56]. Furthermore, hemocyte modifications, such as the development of a swollen or naked nucleus and of condensed chromatin, can be detected 18 h after larvae infection with *Pseudomonas aeruginosa,* and increase over time. In a study by Mizerska-Dudka and Andrejko (2014), [57] the authors showed that hemocytes progression was towards apoptosis, since autophagy-related proteins, such as microtubule-associated protein and caspase, were detected [57]. During the *G. mellonella* seventh larval instar, a reduction in the granulocyte count and an increase in the count of oenocytoids can be observed [52]. Thus, it is strongly recommended that the experiments using the *G. mellonella* model are carried out in the same larval stage.

The humoral response is mediated by soluble immune effector molecules such as opsonins, antimicrobial peptides (AMPs), melanin, extracellular nucleic acids, and products of proteolytic cascades that immobilize or kill the pathogen [58]. Their role will be explained in detail below.

### 3.1. Opsonins 

*Galleria mellonella* produces numerous opsonins, which are hemolymph proteins that identify and attach to conserved microbial elements, namely peptidoglycan recognition proteins (PGRPs), Apolipophorin-III (apoLp-III), hemolin, and *G. mellonella* cationic protein 8 (GmCP8) [59].

PGRPs are identical to mammalian opsonins and bind to peptidoglycan through a conserved domain homologous to a lysozyme of the T4 bacteriophage [60]. 

The apoLp-III is a major exchangeable lipid transport molecule that plays a crucial role in the innate immune response. It has a high affinity for hydrophobic ligands, such as LPS and LTA, and shows a multifunctional role, as it is able to stimulate the phagocytic activity of hemocytes and increase the production of the antimicrobial peptide cecropin [61]. More recently, it was shown that apoLp-III acts synergistically with *G. mellonella* lysozyme, increasing its antimicrobial activity against Gram-negative bacteria [62]. The apoLp-III is also involved in pathogen differentiation, including between Gram-positive and Gram-negative bacteria, and between yeasts and filamentous fungi, and in the establishment of an adequate immune response [63]. The apoLpIII shows high homology with mammalian apolipoprotein E, which is involved in LPS detoxification, and in phagocytosis stimulation [3,64].

Hemolin is a member of the immunoglobulin superfamily, being able to bind to LPS and LTA [65]. Hemolin is expressed in several organs, including the silk gland of the larvae and its fat body, being up-regulated during bacterial and fungal infections [66], or after exposure to low doses of β-1,3-glucan [67]. Both hemolin and GmCP8 stimulate phagocytosis by hemocytes [3,9].

GmCP8 is produced in the fat body, midgut, and integument. It is secreted in the hemolymph and binds firmly to LPS, LTA, and β-1,3-glucan [59]. GmCP8 has activity against bacteria and yeast-like fungi, and this activity is correlated with changes in the microbial cell surface due to the disruption of the membrane integrity [51].

### 3.2. Antimicrobial Peptides

Antimicrobial peptides, or host defense peptides, are usually 10–40 residue polypeptides (although some may have more than one hundred amino acids) produced by virtually all multicellular organisms. They belong to the immune system of all classes of life, providing a broad-spectrum first line of defense against the dissemination of bacteria, virus, protozoa, and fungi [68]. AMPs’ antimicrobial activity results from their cationic charge, which is associated with the presence of lysine, tryptophan, and arginine residues, and from their hydrophobic and amphipathic properties, which allow the peptides to attack the microbial membranes by interacting with their lipids [69,70]. 

Insect AMPs are mainly found in the fat body, hemocytes, digestive tract, salivary glands, and reproductive tract of *G. mellonella*, both in non-infected larvae and in infected larvae, in response to microbial invasion. Some AMPs are identical to molecules characterized in mammals [2,3]. About twenty known or putative AMPs have been identified in *G. mellonella*, namely two cecropins-like peptides, seven moricins, defensins-like peptides, gloverin, *G. mellonella* proline-rich peptides, *G. mellonella* anionic peptides, lysozymes, x-tox, an inducible serine protease inhibitor, heliocin-like peptide, apolipophoricin, and a metalloproteinase inhibitor (IMPI) [2,9].

Cecropins A- and D-like peptides are linear peptides active against Gram-positive and Gram-negative bacteria. Moricins are α-helical peptides that are particularly active against filamentous fungi [71], but also against yeasts and Gram-positive and Gram-negative bacteria [9]. Defensin-like peptides are cysteine-rich cationic peptides that act by forming voltage-dependent ion channels in the cytoplasmic membrane resulting in ion leakage and cell lysis [72]. Defensins include galiomycin (active against filamentous fungi and yeast but without antibacterial activity), *Galleria* defensin gallerimycin (active against entomopathogenic fungi but not active against yeast), and defensin-like peptides (which acts against Gram-positive bacteria and fungi) [72,73]. Gloverin is rich in glycine and it also presents cysteine residues. It inhibits the synthesis of the outer membrane proteins, increasing membrane permeability and showing specific antimicrobial activity [74]. Gm proline-rich peptides 1 and 2 also appear to increase the membrane permeability of bacteria [73]. Gm anionic peptides 1 and 2 bind to positively charged regions of the bacterial membrane and are active against Gram-positive bacteria and fungi [51,73]. Lysozymes are muramidases, able to digest bacterial peptidoglycan and act against Gram-positive bacteria, some Gram-negative bacteria, and some fungi [51,72]. These enzymes are also able to induce apoptotic changes in *Candida albicans* cells [75]. X-tox is an atypical inducible defensin-like peptide that lacks detectable antimicrobial activity [3,76]. The expression of inducible serine protease inhibitor 2, Heliocin-like peptide, and apolipophoricin is induced in response to infection [72,73].

IMPI is the first discovered specific inhibitor of microbial metalloproteinases [77], having zinc in its active center [51,77]. Metalloproteinases are secreted as virulence factors by different human pathogens, being able to destroy insect defense proteins and peptides [78]. Eisenhardt et al. [78] highlighted that IMPI from *G. mellonella* is able to inhibit the elastase and secretome activity of *P. aeruginosa* strains in vitro, and confirmed these results in an in vivo porcine wound model [78]. Insect AMP, such as IMPI directed against extracellular metalloproteinases, could be another strategy to treat bacterial and fungal infections [10].

A study conducted in 2007 [73], which evaluated the antimicrobial activity of cecropin D-like peptide, defensin-like peptide, *Galleria* defensin gallerimycin, Gm proline-rich peptide 1 and 2, Gm anionic peptides 1 and 2, and apolipophoricin against Gram-negative and Gram-positive bacteria, yeast, and filamentous fungi, showed that the most effective molecule was the defensin-like peptide, which was able to inhibit the growth of fungi and susceptible bacteria at small concentrations [3,73]. Recently, it has been reported that *G. mellonella* cecropin A-like peptide exhibits activity against *Escherichia coli*, including biofilm eradication, highlighting the value of insect molecules in the fight against human pathogens [79].

It has recently been shown that AMPs are in part regulated by non-coding microRNAs (miRNAs), as happens in vertebrates [2,80]. Further studies are needed to fully characterize known and putative AMPs produced by *G. mellonella*.

### 3.3. Melanine

Melanization is the synthesis and deposition of melanin around microbes, allowing to encapsulate microbes within the hemolymph, followed by hemolymph coagulation and opsonization [2,35].

The formation of melanin is catalyzed by phenoloxidase (PO), which is produced as the inactive zymogen pro-phenoloxidase (ProPO), an innate immunity protein of cellular and humoral defense, in hemocytes [81]. Melanization begins upon the recognition and phagocytosis of the pathogen, prompting the phenoloxidase pathway. The soluble PRRs coupled to target molecules on bacterial or fungal surfaces trigger the serine protease cascade that results in the cleavage of pro-PO to PO. The activated PO will lead to the non-enzymatic polymerization of quinines to form melanin around microbes and wounds [2,3]. 

The velocity at which *G. mellonella* melanization occurs depends on the virulence of the infecting pathogen [35]. As it starts, the surface of the larval cuticle becomes covered with black spots and, as the infection progresses, it can be completely melanized, leading to the obtaining of completely black and dead larva [3]. A more noticeable melanization occurs in the dorsal region of the larva, the larva’s new immune tissue, where the heart is located [35]. The dorsal region is bordered by sessile hemocytes that sequester the pathogenic agents in the hemolymph, and simultaneously recruit circulating hemocytes to the heart, in which they aggregate and continue phagocytosis [82]. According to Pereira et al. [35], *G. mellonella*’s cellular and humoral immune responses are integrated, because the dorsal region comprises the highest concentration of hemocytes and the greatest melanization, which occurs in the heart [2,35].

Recently, it was suggested that four *G. mellonella* AMPs (defensin-like peptide, proline-rich peptide 1, anionic peptide 2, and lysozyme) have a role in immune modulation, significantly decreasing the activity of hemolymph PO [3,83].

### 3.4. Extracellular Nucleic Acid

Two extracellular nucleic acids are involved in the entrapment and killing of pathogens and in enhancing innate immune responses [84]. Neutrophil extracellular traps (NETs) trap and kill pathogens and are originated by neutrophils that release chromosomal DNA spiked with bactericidal proteins to form NETs upon stimulation with LPS or interleukin-8. Oenocytoids are hemocytes that represent a source of endogenously derived extracellular nucleic acids implicated in both sequestering the pathogen and activating hemolymph coagulation [3,10,84]. 

### 3.5. Influence of Diet on Larval Immune Health 

Several authors already showed that the *G. mellonella* diet can influence its immune system by intervening in larvae development, causing its death, or increasing its susceptibility to infections [1,9,85]. According to Jorjão et al. [1], an energetic diet is associated with a shorter life cycle, increased caterpillar weight, and immune system activation, by enhancing hemolymph volume and a high concentration of hemocytes. This type of diet also lengthens larval survival to bacterial (*S. aureus* and *E. coli*) and fungal (*C. albicans*) infections. In contrast, a weak diet results in a long-life cycle with a short oviposition period and a lengthy egg incubation period [1]. 

Given the influence of diet on the microbiome and the microbiome interaction with the host immune system [41,42,43], it is important to standardize the *G. mellonella* diet or, if necessary, to evaluate its microbiome for a better interpretation of virulence and infection assays outcomes.

## 4. *Galleria mellonella* In Vivo Model—Experimental Design Considerations

Differences in the supplier, breeding conditions, age, weight, nutrition, maintenance, and handling of *G. mellonella* larvae, and the presence of antibiotics and hormones that can alter their metabolism, might easily result in differences in mortality rates generating inconsistent experimental responses in an infection model [9,25]. This might explain conflicting results with some reference strains that induced different larvae mortality rates in studies performed in distinct research labs. The standardization of research protocols parameters would help to provide reliable results and improve inter-laboratory comparisons between results from *G. mellonella* experiments [1,2,10].

Before starting in vivo tests, it is useful to watch some video components of articles done by experienced researchers in order to better understand experimental procedures in *G. mellonella* larva [86,87].

It is recognized that the use of *G. mellonella* reared in the lab is preferable instead of commercially available larvae. Commercial larvae are available from a wide range of independent breeders and are sold as food for reptiles and birds in captivity or as fishing bait. At least ten larvae for each experimental condition should be used in order to reduce the effect of heterogeneity between individuals, and at least two or three independent experiments should be performed. *G. mellonella* should be placed at 25 °C in the dark, from egg to last instar larvae, and fed on a natural diet of beeswax and pollen grains. Individuals selected for experiments should include healthy worms of the final instar larval stage, which develop from the egg in about five weeks, weighing 250 ± 25 mg, having a length of 2–2.5 cm, and a creamy color [3,10,34].

Some authors store larvae at 15 °C before experiments, and starve the larvae for 24 h before infection [87]. Both practices reduce the immune response of *G. mellonella* and thus the virulence of any microorganism tested can be overestimated [85]. Nutrient deprivation has been demonstrated to increase the susceptibility of *G. mellonella* to infection by *C. albicans* [85]. However, these practices can be applied if the study involves pathogenic agents with relatively low virulence [2,85].

Before infection, microbial inoculums should be washed to prevent virulence factors secreted during their in vitro growth from being introduced within the larvae. It is also recommended to have control larvae injected with a placebo inoculum, such as Phosphate Buffer Saline (PBS) or sodium chloride (0.9%), to detect potential physical trauma due to the injection [3,88]. Although most authors use microbial inoculums of 10 µL and 20 µL, smaller microbial inoculum up to 5 µL may also be used [89].

**Figure 1:**
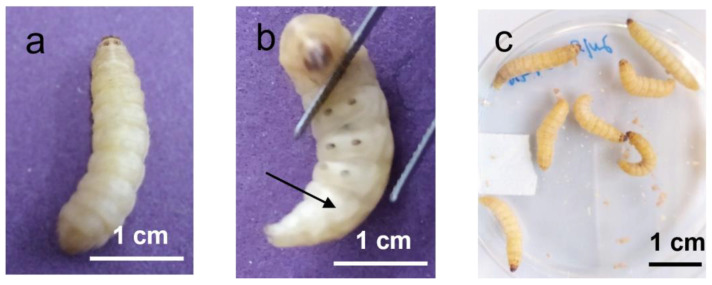
(**a**) Dorsal and (**b**) ventral views of *Galleria mellonella* larva at the last instar stage. The larva is divided into a head, a thorax with three segments, and an abdomen with 10 segments. The segment with the last proleg (arrow) is the fifth, counting from the anal segment with two non-visible anal prolegs; (**c**) Several larvae within a petri dish (original).

The most common infection route is through the hemocoel (the body cavity containing the hemolymph), by inoculation through the last left pro-leg of the abdominal region [90], the surface of which should be previously sanitized with 70% (*v*/*v*) alcohol using a cotton swab. The injection into the last proleg will allow for more space to insert the needle and a fair distribution of the inoculum throughout the body. Further injections e.g., for drug screening, should be given in the opposite upper prolegs (Figure 1).

**Figure 2:**
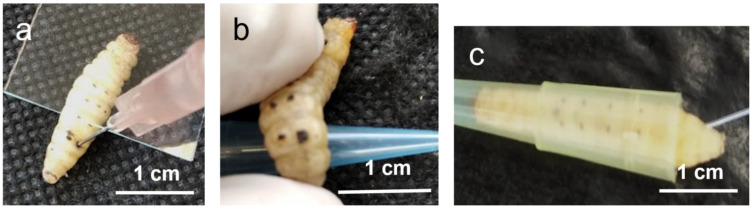
Restraining devices for handling *Galleria mellonella* larva. (**a**) using a microscope blade to immobilize the larva; (**b**) using a blue pipette tip to turn the left proleg to make it more visible; (**c**) using a yellow tip to immobilize the larva (original).

The larva can be immobilized in different ways: by using different restrain devices, such as a microscope blade, a blue pipette tip, or by putting the larva inside a yellow tip [91] (Figure 2); between the fingers of the researcher; or between two sponges, protecting the researcher’s fingers [92]. 

The injection is performed manually, using a fine dosage syringe (up to 1 mL) and a fine needle (up to 0.50 µm), or using an automatic syringe pump which controls the dose injected into each larva. The needle must be inserted parallel to the body so as not to rupture the digestive tract [92].

Oral infection is also a possibility by using a force-feeding method [87], through which the microbial agent is inoculated into the digestive tract and ends up in the intestine with the food bolus. The intestinal microbiota is separated from the bolus by a protecting matrix which prevents the intestinal epithelium from contamination [92]. 

For drug screening, antimicrobial agents can be administered in different treatment regimens, including different times and number of doses. The larvae can be incubated at diverse temperatures that are necessary for the expression of virulence factors [93]. Most studies usually perform the administration of the antimicrobial agent between 30 min to 2 h after infecting the larvae with the pathogen, immediately after infection, or even before infection [3].

Following injection, larvae should be placed in glass Petri dishes and stored in the dark at 37 °C without food, as larvae do not need to eat at the last instar stage [34]. Microbial virulence or antimicrobial evaluation is typically assessed within three to five days. *G. mellonella* can also be used as a model to evaluate photodynamic therapy, because the translucent body of the larva allows light to be rapidly distributed within it, activating the photosensitizer previously injected into the larvae [94]. Larvae display intrinsic autofluorescence, limiting the use of fluorescent proteins to observe the progression of microbial infection over time. Bacterial gene bioluminescence [95] or bacterial RNA expression [96] are viable choices to tackle this issue.

Throughout the assays, the health status of *G. mellonella* should be evaluated using the health index scoring system introduced in 2013 by Loh et al. [97], which evaluates the larvae health status according to the following categories: “survival”, “mobility”, “melanization”, and “cocoon formation” [97] (Table 1). The healthiest larvae are those with the highest score in each category. Thus, the highest score in the “cocoon formation” category should be “no cocoon” instead of “full cocoon”. Therefore, in this review, we propose to alter the scoring of the “cocoon formation” category to “no cocoon”-2, “partial formation”-1, and “full cocoon”-0 (Table 1). This proposal would also allow for the elimination of decimal scoring.

The parameter most frequently evaluated in *G. mellonella* experiments is the survival rate at different time points. The survival curve is the number of larvae that survive as a function of time and is correlated with the virulence of the pathogen or with the effectiveness of antibiotic treatment [3,10,34]. Another parameter to be determined is the number of bacteria or fungi, by counting the colony forming units present in the hemolymph or larvae homogenates over time after culturing on agar medium, or by using bioluminescent microorganisms which can be detected by biophotonic imaging [98]. Using these methods, it is possible to monitor the survival of the pathogen in the larva [3,10,34,98]. Other parameters that can be assessed include the expression of antimicrobial proteins, the production of lactate dehydrogenase as a marker of cell damage, the determination of the medium lethal dose (LD50, which is the dose required to kill half of the larvae tested) [3], the determination of hemocyte density, the evaluation of phenoloxidase activity, the obtaining of histopathological data, the monetarization of bacterial gene expression [10], and of variations in the proteome [9,97].

According to Maslova et al. [99], it is now possible to test compounds in burn wounds by using *G. mellonella*, as burn wounds can be simulated in the larvae by applying a preheated metal to the larval surface [99]. By using scanning electron microscopy, one may observe the main steps of bacterial biofilm genesis on abiotic devices, such as toothbrush bristles, stainless steel, or titanium implants that have been previously indwelled on the larvae surface [100].

Checklist for *Galleria mellonella* experiments:

- It is useful to watch some video components of articles done by experienced researchers.
- It is preferable to use *G. mellonella* reared in the lab instead of commercially available larvae.
- For each experimental condition, at least ten larvae should be used, and at least two or three independent experiments should be performed.
- *G. mellonella* should be placed at 25 °C in the dark, from egg to last instar larvae, and fed on a natural diet.
- Larvae selected for experiments should be healthy worms of the final instar larval stage, weighing 250 ± 25 mg, of 2–2.5 cm long, and with a creamy color.
- Before infection, microbial inoculums should be washed.
- It is recommended to have control larvae injected with a placebo inoculum, such as PBS or sodium chloride (0.9%).
- Use microbial inoculums of 10 µL and 20 µL, or smaller (up to 5 µL).
- The most common infection route: inoculation through the last left pro-leg of the abdominal region, the surface of which should be previously sanitized with alcohol. Further injections e.g., for drug screening, should be given in the opposite upper prolegs (Figure 1).
- The larva can be immobilized by different ways (Figure 2).
- The injection is performed manually using a fine dosage syringe (up to 1 mL) and a fine needle (up to 0.50 µm) or using an automatic syringe pump.
- Oral infection is also a possible method via force-feeding.
- For drug screening: usually the administration of the antimicrobial agent is conducted between 30 min to 2 h after infecting the larvae with the pathogen, or immediately after or before the infection.
- Following injection, larvae should be placed in glass Petri dishes and stored in the dark at 37 °C without food.
- Microbial virulence or antimicrobial evaluation is typically assessed within three to five days throughout by using the health index scoring system, the survival rate at different time points, by counting the colony forming units present in the hemolymph or larvae homogenates over time, or by using bioluminescent microorganisms which can be detected by biophotonic imaging.

## 5. Gram-Positive and Gram-Negative Bacteria Causing Disease in Humans

Studies on Gram-positive bacteria using the in vivo *G. mellonella* model have been performed using several Gram-positive bacteria, *Staphylococcus aureus* being the most frequently used, and Gram-negative bacteria, *Escherichia coli* being the most frequently studied (Table 2). Table 2 shows the total number of publications in Pubmed comprising both the name of the bacterial species or genera and *Galleria mellonella.* These studies do not always use the larva as an infection model, and the number of studies can be overestimated due to the type of Pubmed search made. 

In these studies, different parameters were analyzed to evaluate the effect of bacterial infection on *G. mellonella*, including clinical observations, histopathological analysis, determination of the bacterial burden, the monitorization of immune response activation (hemocytes quantification and viability, the detection of oxygen free radicals, AMP expression, and other markers), the analysis of host miRNA, and bacterial gene bioluminescence and/or bacterial RNA expression.

Human diseases can be caused by polymicrobial infections or by human microbiome dysbiosis. Therefore, detailed studies on the host response during co-infection, including the complex interactions between microbes and also between them and the host, are very important [122].

Two studies on co-infection in cystic fibrosis were performed by using the *G. mellonella* model. The first one comprised a co-infection by the *P. aeruginosa* Liverpool epidemic strain (LES) and Anginosus group streptococci (AGS) [123]. The authors showed that the virulence of LES phenotypic variants can be synergistically enhanced by the presence of oral commensal streptococci, and that this synergy is dependent not only on AGS species but also on the genotype and phenotype of *P. aeruginosa* [123]. The second study focused on a *P. aeruginosa* and *Aspergillus fumigatus* co-infection [124]. It concluded that infecting the larvae with sublethal concentrations of *A. fumigatus* to mimic colonization resulted in significantly higher mortality rates in larvae injected with *P. aeruginosa* 24 h later [124]. Interestingly, Reece et al. [125] noted that *G. mellonella* inoculation with mixed biofilm co-cultures resulted in a reduced biofilm development, and quantification by species-specific qPCR revealed that both pathogens had mutually antagonistic effects on each other [125]. In opposition, Scott et al. [124] found that the presence of *P. aeruginosa* supports *Aspergillus* growth by producing volatile sulfur compounds that provide a sulfur source to the fungus, suggesting that this synergism may increase virulence in co-infection [124].

*Candida albicans* and *S. aureus* are the most common fungal and bacterial agents isolated from bloodstream infections. Sheehan et al. [126] showed that *C. albicans* and *S. aureus* co-infection in a *G. mellonella* model significantly reduced the larval survival relative to the larvae infected with *S. aureus* alone. This was concomitant with infection dissemination, the formation of large nodules, an increase in cecropin D production, and a decrease in apolipophorin, alpha-esterase 45, and serine proteinase, indicating synergism between both species during co-infection [126].

## 6. New Antibacterial Drugs

According to the European Centre for Disease Prevention and Control estimates, more than 35,000 people die from antimicrobial-resistant infections in the European Union/European Economic Area each year [127]. It is concerning that the health impact of antimicrobial resistance is comparable to that of influenza, tuberculosis and human immunodeficiency virus/acquired immunodeficiency syndrome combined [127]. The multidrug resistance hurdle combined with the fact that only two new classes of antibiotics were developed in the last forty years highlight the urgency of finding alternatives to antibiotics [128]. Novel compounds are generally screened in vitro to assess their effectiveness and potential toxicity, and then successful candidates proceed to preclinical trials using animal models, such as *G. mellonella*, before clinical trials in humans [128]. Table 3 shows new antibacterial drugs used alone or as adjuvants to conventional antibiotics, plant derived compounds combined with antibiotics, and combined antibiotics used against human bacterial pathogens tested in *G. mellonella* infection models.

Generally, there is a consistency between in vitro and in vivo results using *G. mellonella* models [3,9,10]. However, some studies show a discrepancy between results. For example, compounds with 1,2,4-triazolidine-3-thione scaffold (an original ligand of the active sites of metallo-β-lactamases, which are important contributors of Gram-negative bacteria resistance to β-lactam antibiotics), proved to be efficient antibacterial agents in in vitro tests, but not in larvae infected with Multidrug resistant (MDR) *Acinetobacter baumannii* AB5075 [130]. Antibiotic combinations against the MDR *P. aeruginosa* strain NCTC13437 showed synergistic effects in a *G. mellonella* model, but there was little correlation with the antibiotic combinations that showed synergy in in vitro tests previously performed [157].

## 7. Antimicrobial Photodynamic Therapy and Phage Therapy

The *Galleria mellonella* model has been used to evaluate the combination of antibiotic therapy and antimicrobial photodynamic therapy (aPDT), which is based on photoactive dye molecules (photosensitizers like methylene blue) that produce reactive oxygen species when irradiated with visible light [3,94]. Treatment with aPTD alone or combined with vancomycin was shown to increase the survival rates of larvae infected with vancomycin-resistant *Enterococcus faecium* when compared to antibiotic treatment alone [94].

Phage therapy is not approved in the United States or Europe, being applied only in rare experimental cases, because antibiotics are more easily available and are considered to be safer [158]. Nevertheless, the *G. mellonella* model was used to assess bacteriophages action against several bacteria such as MRSA [159], vancomycin-resistant enterococcus-infected larvae, KPC-producing *Klebsiella pneumoniae*, MDR *P. aeruginosa*, *P. aeruginosa* isolates from cystic fibrosis patients, carbapenem-resistant Enterobacterales [10,158], and *Burkholderia cenocepacia* strains K56-2 and C6433 [160]. All studies demonstrated an improving survival rate of infected larvae treated with some bacteriophages compared to the phage-free group.

More recently, a phage-antibiotic synergy was described in a *G. mellonella* model, meaning that the efficiency of phage therapy was improved in the presence of sublethal concentrations of certain antibiotics which stimulate lytic phage activity [160,161].

## 8. Fungi Causing Diseases in Humans

The treatment of fungal infections is extremely difficult because of MDR fungal strains which mainly affect immunocompromised patients, and because of a narrow availability of antifungal drugs owing to their high host toxicity [7]. The most studied fungi causing disease in humans are *Aspergillus fumigatus*, *Candida albicans* and *Cryptococcus neoformans*. *A. fumigatus* is the deadliest mold among human pathogenic fungi, with mortality rates of up to 90% being of great medical interest [7,162]. *C. albicans* is the fourth most common cause of hospital-acquired infectious diseases and the primary cause of systemic candidiasis, leading to high mortality rates [163]. *C. neoformans* is associated with morbidity and mortality in immune-deficient patients and transplant recipients [164]. 

Different antifungals, alone or combined with other antifungals or new compounds, were tested against *A. fumigatus* [165], *Aspergillus terreus* [165,166], *C. albicans* [8], *Candida auris* [8], *Candida haemulonii* [167], *Candida tropicalis* [167], *Candida krusei* [167], *Candida lusitaniae* [167], *Candida parapsilosis* and *Candida orthopsilosis* [168], *C. neoformans* [8], the order Mucorales [166], and *Madurella mycetomatis* using *G. mellonella* models [168].

The pharmacokinetics of antifungals can also be examined in *G. mellonella* models by studying the drug increase and distribution in the hemolymph, and drug metabolism and its half-life [168]. Other studies performed using *G. mellonella* models included the characterization of the role of gliotoxin (a sulfur-containing mycotoxin produced by some fungi) in the virulence of *A. fumigatus* [169], of the role of *C. albicans* heat shock protein 90 in antifungal resistance [7], of the importance of certain virulence genes (CAP59, GRA1, RAS1, and PKA1) of *C. neoformans* in the mortality of *G. mellonella* [164], and of the impact of septins (guanosine triphosphate-binding proteins which provide structural support during cell division at the septum) in *C. neoformans* development and virulence at 37 °C [7,168].

*Galleria mellonella* larvae can also be used to assess the in vivo efficacy of antifungal insect defense molecules in crops. Gallerimycin from *G. mellonella* was expressed in tobacco using *Agrobacterium tumefaciens* as a vector, and provided resistance to the fungal pathogens *Erysiphe cichoracearum* and *Sclerotinia minor* [170]. Moreover, derivatives of proteases from *G. mellonella* silk (SPI2) transformed by using *A. tumefaciens* and expressed in potatoes, increased the in vitro resistance of potatoes to late blight (potato blight) *Phytophtora infestans*, while being innocuous to other organisms [171,172].

According to Jackson et al. [173], there are some drawbacks to using insect models to elucidate the *A. fumigatus* pathogenicity in mammals, as this fungal species produces melanin, which is a virulence factor in mammalian models but not in insects. *A. fumigatus* mutants with defects in melanin biosynthesis caused enhanced mortality in larvae following infection, highlighting the importance of studying the innate immunity of the insect [173].

## 9. Toxicity Studies

According to the Global Harmonizing System of Classification and Labelling of Chemicals, the acute toxicity of hazardous chemicals is classified into five categories, ranging from low toxicity (category 5) to high toxicity (category 1) [174]. This classification is based on the LD50 for substances given by oral and dermal routes and on the median lethal concentration of substances administered by the inhalation route that kills half of the tested animals (LC50) [174]. Thereafter, when evaluating the toxicity of an antibacterial or antifungal compounds in the *G. mellonella* model, which is generally administered orally or dermally, LD50 must be evaluated.

*Galleria mellonella* larvae provide a fast and convenient means to assess in vivo toxicity, and results show a strong correlation to those from cell lines, including 3T3 (mouse embryonic fibroblasts) and normal human keratinocytes, and from a murine model [175]. For example, compounds such as doxorubicin (chemotherapy medication used to treat cancer) showed low LD50 values in the *G. mellonella* model similar to that in mammalian models, consistent with its high toxicity [176]. Interestingly, some authors argue that the *G. mellonella* model may even provide more accurate results regarding compounds with low levels of toxicity than cell lines. The use of *G. mellonella* instead of cell lines to ascertain toxicity may prevent low-toxic compounds from being evaluated as toxic and from proceeding to the mammalian model [175].

Several toxicity tests of new compounds with proven in vitro activity towards bacteria or fungi have been evaluated in *G. mellonella*, including antibacterial triazole analogs (one derived from carvacrol and the other derived from 2-hydroxy 1,4-naphthoquinone) bearing carboxylic acid, a series of new bacteriostatic thiourea-containing compounds active towards different gram-positive and gram-negative strains, thiazolylhydrazone derivatives with antifungal activity [9], widely used food preservatives (potassium nitrate, potassium nitrite, potassium sorbate, sodium acetate, sodium benzoate, sodium chloride, sodium nitrate, and sodium nitrite), and imidazole-based ionic liquids widely used in industry [176].

The establishment of the toxic dose of a compound in a *Galleria* model is relatively simple to perform. The first step is to administer the smallest dose of the compound to the larvae. If ≥60% of the larvae survive after five days, the next step is to administer the next higher dose, and so on, until obtaining a toxic dosage [177]. According to Ignasiak et al. [177], compounds that induced >40% mortality following the initial dose may be classified as highly toxic (category 1), whereas those compounds administered at the maximal concentrations that resulted in 100% survival may be classified as non-toxic [176,177]. 

The solvents most widely used for compound testing in *G. mellonella* are PBS or sodium chloride (0.9%), water, and dimethyl sulfoxide (DMSO) (<20%). In a study where the toxicity of several new solvent options were tested in *G. mellonella*, it was established that acetic acid, hydrochloric acid, methanol (MeOH), sodium hydroxide (NaOH) (aqueous solvents), benzyl benzoate, ethyl oleate, isopropyl myristate, and olive oil (non-aqueous solvents) were non-toxic, except for NaOH in concentrations above 0.5 M, and for concentrations of DMSO, MeOH and acetic acid above 30% [178]. Regarding the toxicity of delivery systems, the toxicity of lipid-core nanocapsules coated with micellar layers and a lipophilic crown were evaluated in *G. mellonella,* and the survival rates were similar to those of DMSO and water control samples, being considered non-toxic [179]. This study also showed that *G. mellonella* larvae are potential candidates for toxicity studies of nanomaterials [179]. 

*Galleria mellonella* has a high tolerance to phenolic compounds such as theaflavins and epicatechins, as reported by Betts et al. [180], probably due to its feeding which is based on natural phenolic compounds present in honeycombs [180]. Thus, *G. mellonella* larvae are not the ideal model for the toxicity screening of such compounds, as observed by Lazarini et al. [181], who tested a plant extract (*Eugenia brasiliensis* Lam.) with a high phenol content in *G. mellonella,* observing that it was not possible to determine its LD50, as the compound was non-toxic for the larvae [181].

In preclinical studies, it is important to determine the antibiotic pharmacokinetics and pharmacodynamics to optimize the drug doses in further clinical tests and clinical practice. The mechanisms by which antibiotics are distributed and eliminated in *G. mellonella* are not known [182]. Therefore, the absorption, distribution, metabolism, and excretion (ADME) system of *G. mellonella* is still not completely understood. However, the *G. mellonella* model of infection must enable an antibiotic response likely to be seen in humans. 

There are two published papers whose results firmly support the future application of the *G. mellonella* infection model to initial studies of comprehensive drug candidate analyses [183,184]. A bioassay was validated for fluconazole determination in *G. mellonella* hemolymph, in which an exposure comparable to human exposure was obtained with 20 mg/kg [184]. In addition, there was a clear correlation between measured antibiotic pharmacokinetics, in a range of antipseudomonal antibiotics, and their therapeutic effect against *P. aeruginosa* in *G. mellonella* [183].

## 10. Comparison with Other Invertebrate and Mammalian Models

Traditionally, two other invertebrate models are widely described to study the virulence of bacteria or to test the effect of antimicrobial compounds: *Caenorhabditis elegans* (Phylum nematoda, class Chromadorea) and *Drosophila melanogaster* (Phylum Arthropoda, class of Insects) [7]. The *G. mellonella* model has several advantages over these two models and mammalian models (Table 4), but it is not so well established because it had only started to be widely used more recently [7,10].

*Galleria mellonella* length (between 3–30 mm) is significantly higher than those from *D. melanogaster* and *C. elegans*, (3 mm and 1 mm, respectively), facilitating the operation and handling of the larvae [9,34]. *G. mellonella* can withstand temperatures of 37 °C, unlike the *C. elegans* and *D. melanogaster* models, making it a considerable gain for investigating human pathogens, considering that the expression of many virulence genes is under temperature control [93]. Furthermore, *G. mellonella* exhibits immune phagocytes, unlike *C. elegans,* which is unable to phagocytize microorganisms, conferring on *G. mellonella* a major advantage for the study of several bacteria and fungi [10,25]. *G. mellonella* also shows advantages in studies on the virulence of intracellular bacteria such as *Legionella pneumophila* [86,116], since in the *G. mellonella* model, but not in *C. elegans*, bacteria can penetrate from the intestinal lumen to the intestinal epithelial cells [116]. In addition, the use of *Drosophila* requires more specialized equipment and experience than does *Galleria*, and wild-type *Drosophila* is resistant to fungi [185].

Due to the more recent implementation of the *G. mellonella* model regarding other invertebrate models, there is a lack of stock centers for its commercialization, and of databases such as Flybase (for *D. melanogaster*) or WormBase (*C. elegans*), and only a few microarrays, libraries of RNA interference and mutant strains are available for *G. mellonella* [3,9,10]. Unlike *C. elegans* and *D. melanogaster*, the *G. mellonella* genome (GenBank NTHM00000000) is still a shotgun project that has not been fully analyzed [20]. The lack of mutant and transgenic strains and access to microarrays or RNA interference libraries limit studies on many biological processes and specific diseases. Also, the lack of full genome sequencing and analysis does not allow a detailed molecular study understanding of the host response, and the comparison between genomic, transcriptomic, and proteomic data [10,176]. There is also a lack of standardized experimental procedures or subjective interpretation of scoring parameters, which prevents a direct comparison of different published studies using the *G. mellonella* model [3,9,10]. It is expected that scientific advances will solve these drawbacks soon.

Mammals, such as mouse (Phylum Chordata, Class Mammalia), rabbits and non-human primates, have been used as models to study human pathogens longer than *G. mellonella* [7,10], and for that reason they have gathered a large set of reliable information and to clearly indicate what happens in humans. They also have adaptative immunity, unlike invertebrates [3,4]. Invertebrates are insensitive to pain due to the absence of nociceptors, and consequently there are no restrictive ethical rules [186]. On the other hand, there are ethical concerns and higher costs associated with the in vivo experiments in mammalian models (Table 4). 

Recently, the *G. mellonella* model was successfully used to demonstrate the pathogenicity of the *Mycobacterium tuberculosis* complex [137], showing increased advantages over murine models. Unlike the murine model, which does not normally produce granulomas, a pathognomonic sign of tuberculosis infection, *G. mellonella* produces granulomas after bacterial phagocytosis by the hemolymph [137].

Nonetheless, care must be taken when considering which model is most appropriate to study ubiquitous species, such as *Listeria* spp. [7]. The energy-rich phosphorylated sugar derivatives in invertebrates are an essential source for *Listeria monocytogenes* survival, and invertebrates such as *Galleria* may represent natural reservoirs of the environmental pathogen *Listeria* and other human pathogens [7].

## 11. Conclusions

Research on the insect *Galleria mellonella* was first published in the 1930s, and the larva is now widely used, mainly as a model of infection and for drug screening against bacteria and fungi that cause disease in humans. The model is also used as a toxicity model, and as to study the adjacent immune response in co-infections or in host-pathogen interactions. The limitations of the *Galleria mellonella* model are mainly related to the unavailability of stock centers, commune databases, standardized procedures, mutant and transgenic strains, microarrays or RNA interference libraries, and full genome sequencing and analysis. Regardless, the *Galleria mellonella* model is a virtuous model, because despite having some disadvantages that mainly come from the fact that it has only recently been commonly used, and which we believe will be quickly overcome, its immune system shows remarkable similarities with mammals. Furthermore, there is a positive correlation between the results from *G. mellonella* with mammalian models and with other invertebrate models. Unlike *D. melanogaster* and *C. elegans, G. mellonella* can withstand temperatures of 37 °C, and its handling, as well as microbial inoculation and drug administration, are simpler. In conclusion, *Galleria mellonella* is a valuable in vivo model to be used in preclinical studies as an intermediate model between in vitro and mammalian in vivo studies, and is an excellent example of the application of the bioethics principle of the 3Rs in animal experimentation.

## Figures and Tables

**Table 1:** The *G. mellonella* Health Index Scoring System [97] * and a new score for the “cocoon formation” category proposed.

Category	Status	Score	Proposed Score
Survival	Dead	0	
	Alive	2	
Mobility	no movement	0	
	minimal movement on stimulation	1	
	move when stimulated	2	
	move without stimulation	3	
Melanization	black larvae	0	
	black spots on brown larvae	1	
	≥3 spots on beige larvae	2	
	<3 spots on beige larvae	3	
	no melanization	4	
Cocoon formation	no cocoon	0	2
	partial cocoon	0.5	1
	full cocoon	1	0

* available via license: CC BY.

**Table 2:** Number of publications in Pubmed comprising both the name of the bacterial species or genera and *Galleria mellonella*, and the year of the first publication.

	Number of Publications in Pubmed	First Publication (Year)
Gram-positive bacteria		
*Staphylococcus aureus*	164	2006 [101]
*Bacillus* spp.	111	1968 [102]
*Streptococcus* spp.	62	2000 [103]
*Enterococcus faecalis*	57	2007 [104]
*Listeria monocytogenes*	32	2010 [105]
*Mycobacterium* spp.	29	1952 [106]
*Enterococcus faecium*	21	2009 [107]
Gram-negative bacteria		
*Escherichia coli*	220	1969 [108]
*Pseudomonas aeruginosa*	219	1968 [109]
*Klebsiella pneumoniae*	168	1998 [110]
*Acinetobacter baumannii*	139	2009 [111]
*Burkholderia* spp.	67	2008 [112]
*Salmonella* spp.	44	1968 [15]
*Enterobacter* spp.	34	1991 [113]
*Campylobacter jejuni*	19	2010 [114]
*Yersinia* spp.	19	2002 [115]
*Legionella pneumophila*	12	2012 [116]
*Francisella* spp.	10	2007 [117]
*Clostridium* spp.	10	1972 [118]
*Shigella* spp.	8	2016 [119]
*Porphyromonas gingivalis*	8	2016 [120]
*Helicobacter pylori*	7	2014 [121]

**Table 3:** Screening of antibacterial drugs using *G. mellonella* infection models.

Compounds Tested in In Vivo Model	Microbial Agent
**New antibacterial drugs used alone or as adjuvants to conventional antibiotics**	
Five colistin and lead adjuvants combined, based on a new urea-containing class of 2-aminoimidazole compounds	Highly virulent isolate of *Acinetobacter baumannii* AB5075 resistant to colistin [129]
Compounds with 1,2,4-triazolidine-3-thione scaffold	Multidrug resistant (MDR) *A. baumanni* AB5075 [130]
Combination of new aminoglycoside 6′-*N*-acetyltransferase type Ib inhibitors, which inhibit resistance to aminoglycosides, with amikacin	Amikacin resistant strains *A. baumannii* A155 and *K. pneumoniae* JHCK1 [131]
2-point mutations form of *P. aeruginosa* acyl-homoserine lactone acylase PvdQ (La146W, Fb24Y): a quorum-quenching enzyme	*Burkholderia cenocepacia* [132]
Combination of new 1,2-benzisoselenazol-3(2*H*)-one derivatives (a New Delhi metallo-ß-lactamase-1 (NMD-I) inhibitor) and meropenem	Carbapenem resistant *Enterobacter cloacae* producing NDM-1 [133]
Estrogen receptor antagonist tamoxifen	*Enterococcus faecium* 824-05 [134]
Gold(I) pioneer complexes bearing *N*-heterocyclic carbenes and steroid derivatives (ethynylestradiol and ethisterone)	*Escherichia coli* [135]
Zinc chelator: zincophore (S,S)-ethylenediamine-*N,N*′-disuccinic acid (EDDS), alone or combined to imipenem	*Klebsiella pneumoniae* producing NMD-I [136]
Antituberculosis drugs (isoniazid, rifampicin, pyrazinamide, ethambutol, and moxifloxacin)	*Mycobacterium tuberculosis* Complex [137]
Macrolide (azithromycin) with a new synthetic adjuvant (a bis-2-aminoimidazole derivative) in combination with azithromycin	Macrolide resistant *Pseudomonas aeruginosa* laboratory strain PAO1 [138]
New amphiphilic tobramycin-lysine conjugates alone and in association with minocycline and rifampicin	Extensively drug-resistant *P. aeruginosa* P262 [139]
Tobramycin−moxifloxacin hybrid core structure (moxifloxacin is linked via a C12-tether to the C-5 position of tobramycin)	MDR *P. aeruginosa* 104354 (resistant to all classes of antipseudomonal agent except colistin) [140]
Antimicrobial peptide LL-37 (and the enantiomer D-LL-37)	*P. aeruginosa* ATCC19429 [141]
Non-ribosomal tobramycin-cyclam conjugate with meropenem, aztreonam, or ceftazidime/avibactam	MDR *P. aeruginosa* [142]
New bioactive compound (SKC3) isolated from marine sponge-derived *Streptomyces* spp. SBT348	*Staphylococcus aureus* [143]
A novel protonophore 1-(4-chlorophenyl)-4,4,4-trifluoro-3-hydroxy-2-buten-1-one	MRSA strain MW2 [5]
Antihistamine terfenadine	*S. aureus* UAMS-1112 [134]
Carbene silver(I) acetate derivative (SBC3)	*S. aureus* [144]
Metal ions (silver and zinc)	MRSA [145]
Tetrameric-iodine and polymeric silver complexes of the omeprazole scaffold	*C. albicans*, *E. coli*, *S. aureus* and *P. aeruginosa* infections [146]
**Plant derived compounds combined with antibiotics**	
Baicalin hydrate (flavonoid from the roots of *Scutellaria baicalensis*) and cinnamaldehyde (isolated from cinnamon oil) alone or combined with tobramycin	*B. cenocepacia* [147]
*N*-phenyl-1*H*-pyrazole-4-carboxamide derivatives and other pyrazoles (from e.g., *Rhizophora apiculata*)	*S. aureus* ATCC 29213 [148]
Antibiofilm compounds hamamelitannin (polyphenol extracted from the bark of *Hamamelis virginiana*, is a quorum-sensing (QS) inhibitor) alone or combined with clindamycin or vancomycin	*S. aureus* [147]
*Eugeria brejoensis* essential oil (flavonoid) alone and combined with ampicillin, chloramphenicol, or kanamycin	*S. aureus* [149]
Myricetin (flavonoid found in plants like the strawberry and spinach)	*S. aureus* [150]
**Combined antibiotics**	
Imipenem-colistin	MDR *E. cloacae* [151]
Gentamicin and daptomycin	Vancomycin-sensitive *E. faecalis* or vancomycin-resistant *Enterococcus faecium* [152]
Linezolid plus fosfomycin	Vancomycin-resistant *E. faecium* [153] *S. aureus* [154]
Oritavancin, a new glycopeptide vancomycin-derivative antibiotic, with an extended half-life span alone VS ceftriaxone, gentamicin and daptomycin alone or combined	Vancomycin-susceptible and vancomycin-resistant enterococci strains [155]
Ceftazidime/avibactam alone at high doses, or combined with other antibiotics (polymyxin B, amikacin or meropenem)	Klebsiella pneumoniae carbapenemase (KPC)-producing *K. pneumoniae* strains resistant to ceftazidime/avibactam [156]
Azithromycin, ciprofloxacin, levofloxacin and streptomycin	Live vaccine strain of *Francisella tularensis* [117]
Dual combinations of cefotaxime and piperacillin, amikacin and meropenem, or the triple combination of piperacillin, amikacin and meropenem	MDR *P. aeruginosa* strain NCTC13437 [157]

MDR, Multidrug resistant; NMD-I, New Delhi metallo-β-lactamase-1; MRSA, Methicillin-resistant *Staphylococcus aureus*; KPC, *Klebsiella pneumoniae* carbapenemase.

**Table 4:** Comparison between major invertebrate and mammalian models.

Features	*C. elegans* (Nematode)	*D. melanogaster* (Arthropoda)	*G. mellonella* (Arthropoda)	*Mus musculus* (Chordata)
Animal size	Small	Small	Significant	Large
Specialized experience needed	No	Yes	No	Yes
Stock centers	Yes	Yes	No	Yes
Commune databases	Yes	Yes	No	Yes
Standardized procedures	Yes	Yes	No	Yes
Mutant and transgenic strains available. Access to microarrays or RNA interference libraries	Yes	Yes	No	Yes
Full genome sequencing and analysis	Yes	Yes	No	Yes
Adaptative immunity	No	No	No	Yes
Innate immunity	Yes	Yes	Yes	Yes
Phagocytosis by immune cells	No	Yes	Yes	Yes
Invasion of intestinal epithelial cells by bacteria	No	Yes	Yes	Yes
Survival at 37 °C	No	No	Yes	Yes
Cost-effective	Yes	Yes	Yes	No
Ethical concerns	No	No	No	Yes

## Data Availability

No new data were created.
